# A case of *Magnusiomyces capitatus* isolated during monitoring in an antimicrobial diagnostic stewardship context.

**DOI:** 10.1016/j.idcr.2024.e01959

**Published:** 2024-04-15

**Authors:** Francesco Foglia, Giuseppe Greco, Carla Zannella, Annalisa Chianese, Annalisa Ambrosino, Alessandra Conzo, Giovanni Conzo, Anna De Filippis, Emiliana Finamore, Ludovico Docimo, Massimiliano Galdiero

**Affiliations:** a Department of Experimental Medicine, Section of Microbiology and Clinical Microbiology, University of Campania "Luigi Vanvitelli", 80138 Naples, Italy; b Complex Operative Unity of Virology and Microbiology, University Hospital of Campania "Luigi Vanvitelli", 80138 Naples, Italy; c Division of General and Oncologic Surgery, Department of Cardiothoracic Sciences, University of Campania "Luigi Vanvitelli", Via Pansini 1, 80131 Naples, Italy

**Keywords:** *Magnusiomyces capitatus*, Antimicrobial Diagnostic Stewardship, Surveillance, Yeast infection

## Abstract

*Magnusiomyces capitatus (M. capitatus)* is an emerging opportunistic yeast in the Mediterranean region typically isolated from immunocompromised patients, usually affected by blood malignancies. We reported a rare case of *M. capitatus* infection, isolated from a drainage fluid in a patient affected by lung cancer recovered in the University Hospital of Campania “Luigi Vanvitelli”, Naples, Italy. The isolate was identified by phenotypic methods, i.e., Gram and Lactophenol cotton blue (LCB) staining, and matrix-assisted laser desorption ionization–time of flight mass spectrometry (MALDI-TOF MS) analysis. We identified *M. capitatus* on the third day from Sabouraud Dextrose Agar supplemented with chloramphenicol and gentamicin. Antifungal susceptibility test revealed that 5-fluorocytosine was the most active drug against *M. capitatus*, followed by itraconazole and voriconazole, micafungin, amphotericin B and fluconazole, posaconazole, anidulafungin, and caspofungin. Our data showed the importance of an early cultural and fast microbiology diagnosis based on the characteristic morphologic features observed in Gram-stained smears of blood culture positive bottles, and the validation via MALDI-TOF MS. This dual approach has significant impact in the clinical management of infectious diseases and antibiotic stewardship, by integrating sample processing, fluid handling, and detection for rapid bacterial diagnosis.

## Introduction

In recent years, fungal infections represent a huge and emergent worldwide public health problem with high financial costs for diagnosis, treatment, and prevention [1], [2]. The severity of the disease varies from bland or asymptomatic skin to appendages or lethal systemic infections [3]. Numerous cases are not correctly diagnosed, and turnaround time of conventional diagnostic methods limit the detection of superficial fungal infections probably underestimated. In addition, changes of pathogen distribution, demographics, and antimicrobial resistance in according with antimicrobial diagnostic stewardship program, may affect the epidemiology. The development and optimization of rapid diagnostic approaches for the identification of bacteria represents one of the priorities not only to combat antimicrobial resistance, but also to lead a more accurate detection of pathogens that typically occur at low concentrations in biological samples or are uncommon. Therefore, an accurate etiological diagnosis that combines phenotypic methods, including Gram staining, culture and biochemical test, to molecular analyses, is important both for the prescription of the correct therapeutic treatment [4], [5] and for prevention programs [6].

*Magnusiomyces capitatus* (*M. capitatus*), previously known as *Geotrichum capitatum*, *Dipodascus capitatus*, *Trichosporon captiatum*, *Saprochaete capitata*, or *Blastoschizomyces capitatus*, is an emerging yeast with intrinsic resistance to many commonly used antifungal agents [7], [8]. Systemic infections have been described such as endocarditis, fungemia, and pulmonary infections, especially in patients with hematological disorders [9].

Here we report a case of isolation of *M. capitatum* in an immunocompromised patient with a solid cancer and resection of the upper pulmonary lobe recovered in the University Hospital of Campania “Luigi Vanvitelli”, Naples, Italy.

We describe the important role of antimicrobial diagnostic stewardship program used in our hospital which can support correct treatment strategies.

## Case presentation

An 80-year-old male with an anamnesis of juvenile exanthematous diseases, hypertension (in treatment), bronchopneumopathie chronique obstructive (BPCO) and hyperthyroidism (in treatment), hematemesis episodes treated with transfusions, and a malignant tumor which led to the segmental resection of the upper pulmonary lobe in 2019, was successively subjected to duodenocephalopancreasectomy. His post-operative course was complicated by fever (38,5 °C), sinking of the pancreatic stump and anastomosis of gastrojejunum and hepatojejunum. Therefore, he has been hospitalized a month later in July 2022 showing a duodenal heteroplasia in gastroscopy. The patient was subjected on screening for Hepatitis B surface antigen (HbsAg) and Hepatitis C virus antibody (HCVAb) on the first day of hospitalization and both showed negative results. However, capillary serum protein electrophoresis reported increased values of alpha-1, alpha-2 and gamma-globulins, and also decreased levels of albumin, albumin/globulin ratio (A/G) and total proteins. On the contrary, beta-1 and beta-2 globulins showed normal parameters. A month later the patient showed symptoms of infection and drainage was performed, and a part of the drainage liquid was collected on day 28 of hospitalization.

**Fig. 1:**
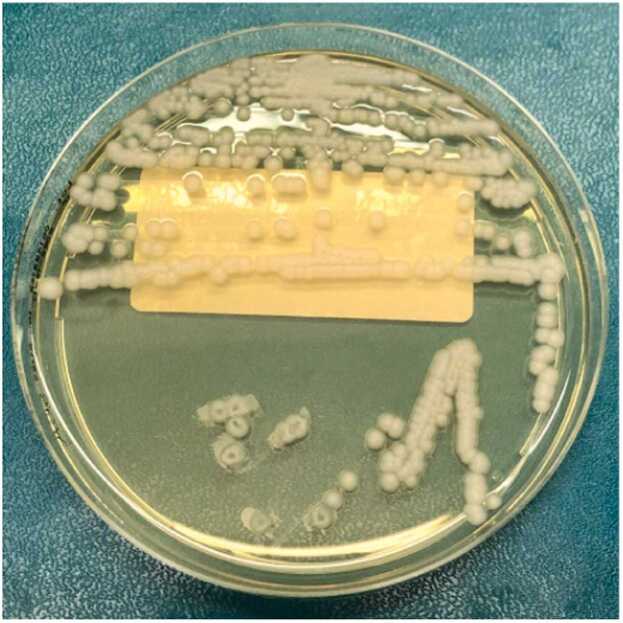
*M. capitatus* on Sabouraud CAF GEN agar plate.

It was rapidly received in our Complex Operative Unit (UOC) of Virology and Microbiology, where it was seeded both on agar plates and in Brain Heart Infusion (BHI) broth, then incubated at 37 °C overnight. On the first day, *Escherichia coli* (*E. coli*) colonies grew up, while *M. capitatus* appeared on the third day in Sabouraud Dextrose Agar supplemented with chloramphenicol and gentamicin (Sabouraud CAF GEN) (Fig. 1). In detail, three different sets, each consisting of two flasks collected in three different days, were performed; all were positive for *E. coli*, and two out of the three were positive for *M. capitatus*.

**Fig. 2:**
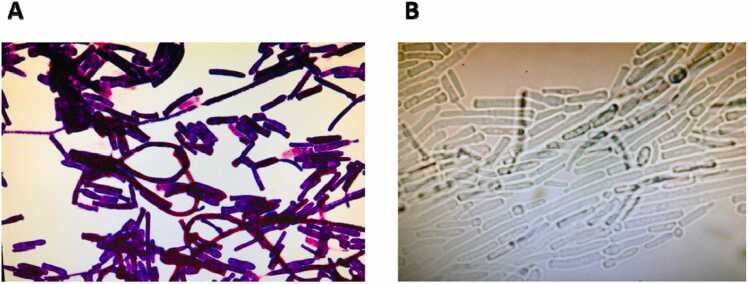
**(A)** Gram-stained smears from positive blood cultures with typical arthroconidial forms of *M. capitatus*. Magnification, x 100. **(B)** LCB staining of *M. capitatus.*

In addition, Gram staining from positive drainage cultures showed typical arthroconidial forms with purple color (Fig. 2 A). Then, a Lactophenol cotton blue (LCB) staining was performed identifying the presence of fungi with characteristic filamentous form (Fig. 2B).

**Fig. 3:**
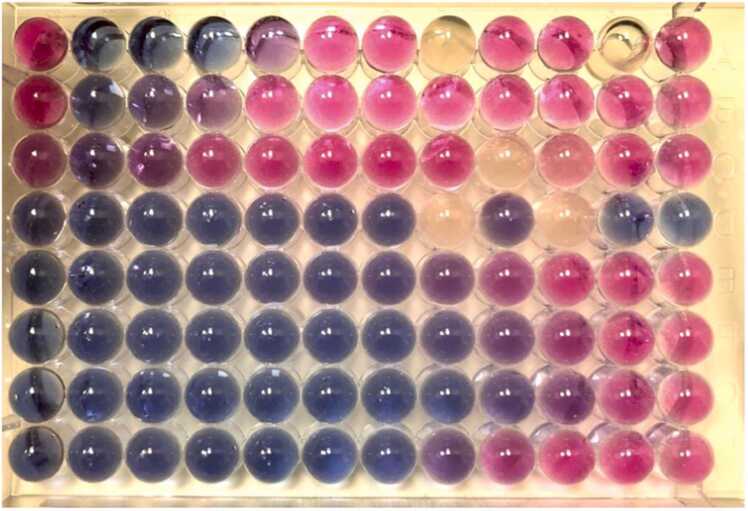
Antimicogram of *M. capitatus.* Anidulafungin: from 0.015 to 8 μg/mL; amphotericin B: from 0.12 to 8 μg/mL; micafungin: from 0.008 to 8 μg/mL; caspofungin: from 0.008 to 8 μg/mL; 5-flucytosine: from 0.06 to 64 μg/mL; posaconazole: from 0.008 to 8 μg/mL; voriconazole: from 0.008 to 8 μg/mL; itraconazole: from 0.015 to 16 μg/mL; fluconazole: from 0.12 to 256 μg/mL. *Candida parapsilosis* ATCC 22019 and *Candida krusei* ATCC 6258 were used as quality control strains.

Microorganisms identification was performed by matrix-assisted laser desorption ionization–time of flight mass spectrometry (MALDI-TOF MS) by using Bruker Microflex instrument, Biotyper software (Version 3.0), and database (Version 3.1.66; Bruker Daltonics). The identification species-related data generated by MALDI-TOF MS were classified following the manufacturer’s instructions. The score values ≥ 2.0, 1.7–1.99, and < 1.7, indicated species identification, genus level detection, and Non-Reliable Identification (NRI), respectively. The score provided by MALDI-TOF in both isolations, and repeated up to 4 times, was > 2, thus confirming the species as *M. capitatus* (>99% confidence rate). Spectra were baseline corrected and normalized to total positive ion current [10]. Antibiogram was performed in automatized manner showing an Extended-Spectrum Beta-Lactamases (ESBL) producing *E. coli* sensible for piperacillin-tazobactam and cephalosporins with Beta-Lactamases inhibitors and the patient was treated for thisinfection. However, no treatment was decided to be applied for the *M. capitatus* infection. As reported in Fig. 3, in vitro antifungal susceptibility of *M. capitatus* was tested by Sensititre microdiluition (Thermo Fisher Scientific) and in Table 1 the relative minimal inhibitory concentration (MIC) values were indicated.

**Table 1:** MIC of *M. capitatus.*

**Antimycotic drug**	**MIC**
Anidulafungin	2 μg/mL
Micafungin	8 μg/mL
Caspofungin	8 μg/mL
5-flucytosine	0.008 μg/mL
Posaconazole	0.25 μg/mL
Voriconazole	0.12 μg/mL
Itraconazole	0.12 μg/mL
Fluconazole	1 μg/mL
Amphotericin B	1 μg/mL

5-fluorocytosine, fluconazole, itraconazole, posaconazole, voriconazole, caspofungin, amphotericin B, anidulafungin, and micafungin, were used according to the US Clinical and Laboratory Standards Institute (CLSI M27-A4, 2008), with *Candida parapsilosis* ATCC 22019 and *Candida krusei* ATCC 6258 used as quality control strains. Aliquots of 100 μl of the diluted sample and the positive controls were inoculated into the wells with antifungals. The plate was incubated at 28 °C until a change in color from blue (indicative of no growth) to red (indicative of growth). When a purple color remained during a change from red to blue (indicative of partial growth inhibition), the MIC was defined as the lowest drug concentration which resulted in a purple color.

The results showed that 5-fluorocytosine was the most active drug against *M. capitatus* with the lowest MIC at 0.008 μg/mL, followed by itraconazole and voriconazole (0.12 μg/mL), micafungin (8 μg/mL), amphotericin B and fluconazole (1 μg/mL), posaconazole (0.25 μg/mL), anidulafungin (2 μg/mL), and caspofungin (8 μg/mL).

## Discussion

Currently, blood culture represents the gold standard for diagnosis of fungemia or candidemia. It has the advantage of isolating the etiologic agent at species level and performing susceptibility testing. Nevertheless, blood culture is characterized by a long turnaround time since on average, it takes around 24–48 h from positive blood culture to the identification of the species.

*M. capitatus* is an ascomycetous yeast known to cause life-threatening invasive infections in immunocompromised patients, particularly in the case of hemato-oncological malignancies [11], [12], [13] In detail, infections with these species are associated with a high mortality rate ranged from 40 to 80% [14], [15]. In recent years, the numbers of published cases of infections with *Magnesiomyces clavatus* (*M. clavatus*) and *M. capitatus* have increased, and both species are considered as emerging pathogens [16], [17].

The correct identification of *M. capitatus* is challenging, because *M. clavatus* is frequently misidentified as *M. capitatus* by biochemical identification methods [18], [19], [20].

Evaluating the patient’s outcome, the isolation of *M. capitatus* from the drainage liquid was incidental more than suggestive for a coinfection, however considering the sample from which it has been isolated and the relative rarity of the strain itself, we considered significant to highlight this isolation considering the emerging status of *M. capitatus* as a potential pathogen. Currently, no clinical breakpoints or therapeutic guidelines are available for treating *M. capitatus* infection. By MIC analysis, we observed a sensibility to azol and 5-flucytosine, but resistance to anidulafungin, micafungin, and caspofungin.

In this case, we highlighted the value of direct microscopy in diagnosing *Magnusiomyces* bloodstream infection, since we observed the characteristic microscopic feature characterized by arthroconidial forms. It can offer a distinct advantage in achieving rapid diagnosis and may help clinicians to choose an appropriate antifungal therapy.

## Conclusions

This case remarks the importance of a careful surveillance of uncommon fungal infections and the necessity to elaborate and establish valuable breakpoints to determine both sensibility and resistance of antifungals. It has to be strongly associated with the requirement of an Antimicrobial Diagnostic Stewardship among labs and hospitals in order to improve both therapeutic treatments and outcomes. In addition, this case describes the value of Gram-stained smear from positive blood cultures in the early presumptive diagnosis of *M. capitatus* fungemia.

## Funding

Any founding supported this work.

## Ethical approval

All methods used in the study were in accordance with the international guidelines.

## Authors contribution

F.F. participated in data collection and analysis and wrote the first draft of the manuscript; G.G. participated in data analysis; C.Z., A.C. and A.A. performed the literature review; A.Co. and G.C. cured the data; A.D.F. and E.F. contributed to the review of the final version; M.G. contributed to the supervision and project administration.

## CRediT authorship contribution statement

**Alessandra Conzo:** Data curation. **Annalisa Ambrosino:** Methodology. **Annalisa Chianese:** Methodology. **Carla Zannella:** Methodology. **Giuseppe Greco:** Software. **Francesco Foglia:** Writing – original draft, Formal analysis, Conceptualization. **Massimiliano Galdiero:** Validation, Supervision. **Ludovico Docimo:** Supervision. **emiliana finamore:** Writing – review & editing. **Anna De Filippis:** Writing – review & editing. **Giovanni Conzo:** Data curation.

## Declaration of Competing Interest

The authors declare that they have no known competing financial interests or personal relationships that could have appeared to influence the work reported in this paper.

## References

[1] Drgona L., Khachatryan A., Stephens J., Charbonneau C., Kantecki M., Haider S. (2014). Clinical and economic burden of invasive fungal diseases in Europe: focus on pre-emptive and empirical treatment of Aspergillus and Candida species. Eur J Clin Microbiol Infect Dis.

[2] Almeida F., Rodrigues M.L., Coelho C. (2019). The still underestimated problem of fungal diseases worldwide. Front Microbiol.

[3] Badiee P., Hashemizadeh Z. (2014). Opportunistic invasive fungal infections: diagnosis & clinical management. Indian J Med Res.

[4] Riwes M.M., Wingard J.R. (2012). Diagnostic methods for invasive fungal diseases in patients with hematologic malignancies. Expert Rev Hematol.

[5] Arvanitis M., Anagnostou T., Fuchs B.B., Caliendo A.M., Mylonakis E. (2014). Molecular and nonmolecular diagnostic methods for invasive fungal infections. Clin Microbiol Rev.

[6] Foglia F., Della Rocca M.T., Melardo C., Nastri B.M., Manfredini M., Montella F. (2022). Bloodstream infections and antibiotic resistance patterns: a six-year surveillance study from southern Italy. Pathog Glob Health.

[7] D'Assumpcao C., Lee B., Heidari A. (2018). A case of Magnusiomyces capitatus Peritonitis without underlying malignancies. J Invest Med High Impact Case Rep.

[8] Noster J., Koeppel M.B., Desnos-Olivier M., Aigner M., Bader O., Dichtl K. (2022). Bloodstream infections caused by Magnusiomyces capitatus and Magnusiomyces clavatus: epidemiological, clinical, and microbiological features of two emerging yeast species. Antimicrob Agents Chemother.

[9] Mazzocato S., Marchionni E., Fothergill A.W., Sutton D.A., Staffolani S., Gesuita R. (2014). Epidemiology and outcome of systemic infections due to saprochaete capitata: case report and review of the literature. Infection.

[10] Ercibengoa Arana M., Alonso M., Idigoras P., Vicente D., Marimon J.M. (2018). Matrix-assisted laser desorption ionization-time of flight mass spectrometry (MALDI-TOF) score algorithm for identification of Gordonia species. AMB Express.

[11] Durán Graeff L., Seidel D., Vehreschild M.J.G.T., Hamprecht A.G., Kindo A.J., Ráčil Z. (2017). Invasive infections due to Saprochaete and Geotrichum species: Report of 23 cases from the FungiScope Registry. Mycoses.

[12] Menu E., Criscuolo A., Desnos-Ollivier M., Cassagne C., D’Incan E., Furst S. (2020). Saprochaete clavata outbreak infecting cancer center through dishwasher. Emerg Infect Dis.

[13] Seidel D., Duran Graeff L.A., Vehreschild M., Wisplinghoff H., Ziegler M., Vehreschild J.J. (2017). FungiScope(.) -Global emerging fungal infection registry. Mycoses.

[14] Lo Cascio G., Vincenzi M., Soldani F., De Carolis E., Maccacaro L., Sorrentino A. (2020). Outbreak of saprochaete clavata sepsis in hematology patients: combined use of MALDI-TOF and sequencing strategy to identify and correlate the episodes. Front Microbiol.

[15] El Zein S., Hindy J.R., Kanj S.S. (2020). Invasive saprochaete infections: an emerging threat to immunocompromised patients. Pathogens.

[16] Buchta V., Bolehovska R., Hovorkova E., Cornely O.A., Seidel D., Zak P. (2019). Saprochaete clavata invasive infections - a new threat to hematological-oncological patients. Front Microbiol.

[17] Ulu-Kilic A., Atalay M.A., Metan G., Cevahir F., Koc N., Eser B. (2015). Saprochaete capitata as an emerging fungus among patients with haematological malignencies. Mycoses.

[18] Del Principe M.I., Sarmati L., Cefalo M., Fontana C., De Santis G., Buccisano F. (2016). A cluster of Geotrichum clavatum (Saprochaete clavata) infection in haematological patients: a first Italian report and review of literature. Mycoses.

[19] Desnos-Ollivier M., Blanc C., Garcia-Hermoso D., Hoinard D., Alanio A., Dromer F. (2014). Misidentification of Saprochaete clavata as Magnusiomyces capitatus in clinical isolates: utility of internal transcribed spacer sequencing and matrix-assisted laser desorption ionization-time of flight mass spectrometry and importance of reliable databases. J Clin Microbiol.

[20] Hamprecht A., Christ S., Oestreicher T., Plum G., Kempf V.A., Gottig S. (2014). Performance of two MALDI-TOF MS systems for the identification of yeasts isolated from bloodstream infections and cerebrospinal fluids using a time-saving direct transfer protocol. Med Microbiol Immunol.

